# Interaction of discourse processing impairments, communicative participation, and verbal executive functions in people with chronic traumatic brain injury

**DOI:** 10.3389/fpsyg.2022.892216

**Published:** 2022-09-26

**Authors:** Julia Büttner-Kunert, Sarah Blöchinger, Zofia Falkowska, Theresa Rieger, Charlotte Oslmeier

**Affiliations:** ^1^Department of Linguistics, Project NEUROPRAG, Ludwig Maximilians University, Munich, Germany; ^2^Department of Linguistics, Speech-Language-Therapy, Ludwig Maximilians University, Munich, Germany; ^3^Speech-Language Therapy Unit, NEUROKOM, Bad Tölz, Germany

**Keywords:** traumatic brain injuries, communication disorder, executive function, communicative participation, verbal fluency, narration, MAKRO-Screening, La Trobe Communication Questionnaire (LCQ)

## Abstract

**Introduction:**

Especially in the chronic phase, individuals with traumatic brain injury (TBI) (IwTBI) may still have impairments at the discourse level, even if these remain undetected by conventional aphasia tests. As a consequence, IwTBI may be impaired in conversational behavior and disadvantaged in their socio-communicative participation. Even though handling discourse is thought to be a basic requirement for participation and quality of life, only a handful of test procedures assessing discourse disorders have been developed so far. The MAKRO Screening is a recently developed screening tool designed to assess discourse impairments. The test construction is based on psycholinguistic frameworks and the concept of *macro-rules*, which refer to cognitive functions responsible for organizing and reducing complex information (e.g., propositional content) in discourse.

**Aim:**

The aim of our study was to investigate discourse processing in IwTBI in different tasks and to assess problems in communicative participation in the post-acute and chronic phase. In this context, we also aimed to analyze the influence of the severity of the initial impairment and the verbal executive abilities on the discourse performance. Additionally, the impact of macrolinguistic discourse impairments and verbal fluency on perceived communicative participation was targeted in our analysis.

**Methods:**

Data from 23 IwTBI (moderate to severe) and 23 healthy control subjects have been analyzed. They completed two subtests of the MAKRO screening: *Text production* and *Inferences*. Discourse performance was examined in relation to measures of semantic fluency and verbal task-switching. Socio-communicative problems were evaluated with the German version of the La Trobe Communication Questionnaire (LCQ).

**Results:**

IwTBI showed lower test results than the control group in the two subtests of the MAKRO-Screening. Difficulties in picture-based narrative text production also indicated greater perceived difficulties in communicative participation (LCQ). We also found that the subject’s performance on the MAKRO-Screening subtests can partly be explained by underlying dysexecutive symptoms (in terms of verbal fluency and verbal task switching) and the severity of their injury. The preliminary results of our study show that cognitive-linguistic symptoms in IwTBI are also evident in the chronic phase. These can be detected with procedures referring to the discourse level, such as the MAKRO-Screening. The assessment of discourse performance should be an integral part in the rehabilitation of IwTBI in order to detect cognitive-linguistic communication disorders and to evaluate their impact on socio-communicative participation.

## Introduction

With a few exceptions, the importance of cognitive influences on neurogenic language disorders in traumatic brain injury (TBI) has been understudied in aphasiology research (Heilman et al., 1971; Grochmal-Bach et al., 2009; Büttner, 2017). One early proposal by Luria (1970) described trauma-related language disorders as impairments to a network of “language activity” and “disturbances of thought processes” due to lesions to the frontal lobe, resulting in communication disorders “at the borderline between aphasia and disturbances of thought” (p. 208). Verbal planning difficulties at the discourse level result from both the ability to select thematic-relevant propositions and the syntagmatic organization of language necessary for building a coherent (narrative) macrostructure. Recent findings have confirmed Luria’s works concerning the influence of executive functions (EFs), which are located in the prefrontal cortex, and have carried on with his seminal work on the cognitive-linguistic interaction at the discourse level (Alexander, 2002; Ferstl et al., 2008; Ardila et al., 2020). Importantly, a large number of imaging studies and meta-analyses support the view of an “extended language network” (Ferstl et al., 2008) including specific modules processing context-specific language or natural language use beyond traditional language areas and pathways (Mason and Just, 2006; Coelho et al., 2013; Hertrich et al., 2020). For instance, AbdulSabur et al. (2014), show that both narrative production and comprehension include areas associated with the ability to construct situation models and to understand and reflect on the mental states of oneself and others, like the dorsomedial prefrontal cortex, the precuneus, inferior parietal regions or the premotor cortex.

Pathomechanisms of focal and diffuse injuries in TBI lead to a disruption between cognitive and linguistic abilities, making this pathology an ideal candidate for examining the linguistic-cognitive interplay (Snow et al., 1997; Büttner, 2016).

In the International Classification of Functioning, Disability and Health (ICF) of the World Health Organisation (WHO), TBI can be described as impairments of body structures (s110, structure of the brain). Impairments of functional ability due to a TBI can be coded as, for example, higher-level cognitive functions (b164) or as mental functions of language (b167). The consequences for the levels of activity and participation are manifold and can be described, for example, under d175 solving problems, d350 conducting conversations or d670 family relationships (DIMDI, 2012; Laxe et al., 2013).

TBIs can be caused by direct or indirect blows (e.g., blast waves from explosion) to the head (Rosenfeld et al., 2013). This can result in both covered and open brain injuries, which are mostly caused by falls or accidents as a result of violence to the head, with consequences such as skull fractures, contusion, intracranial bleeding, or ischemia (Maegele et al., 2019). Due to coup-contre-coup mechanisms, focal injuries to the frontal and temporal poles are common (Drew and Drew, 2004). TBI can also result in shearing injuries to the nerve fibers, or white matter which are also known as *diffuse axonal injury* (DAI) (McDonald et al., 2014; Raukola-Lindblom et al., 2021). DAI can severely impair functions of the dorso-lateral prefrontal cortex (DLPFC) as well as the corpus callosum (Raukola-Lindblom et al., 2021). The Glasgow Coma Scale (GCS) is the most widely used system for classifying the severity of TBI. It ranks a person’s state of consciousness on a scale of 3 to 15 based on verbal, motor and eye responses to stimuli immediately after the time of the trauma. Mild TBI is rated from 13 to 15, moderate from 9 to 12 and severe from 3 to 8 on the GCS. Despite its widespread use, classification using the GCS has been criticized because the timing of the assessment of the initial state of consciousness varies. The length of altered state of consciousness seems to be a more reliable index to determine severity. This phase of confusion immediately following a coma has been termed post-traumatic amnesia (PTA). According to the severity classification using PTA, a patient with a period of altered consciousness <1 day is classified as mild. A period of altered consciousness lasting 1–7 days is classified as moderate and >7 days as severe (McDonald et al., 2014). Although the presence of PTA in particular is considered as a good index of outcome after TBI, the methods to detect PTA are inconsistent (prospective vs. retrospective methods) and the distinction from chronic memory impairment is not always clear (Tate and Pfaff, 2000). Therefore, whenever possible, several different assessment methods are reported for the overall impression of TBI severity.

Cognitive impairment in TBI can be caused by either direct lesion of fronto-temporal brain areas or by disruption of cortico-subcortical networks (Ardila, 2012). In particular, DAI involving the DLPFC is associated with impaired EFs (Lipton et al., 2009). Heterogenous dysfunctions can emerge in the form of impairments in attention or working memory and disturbances in organizing and monitoring actions, but also as disorders in cognitive flexibility and social cognition (Ylvisaker and DeBonis, 2000; Mcdonald et al., 2014). Disturbances of EFs can affect the organization and control of basic cognitive abilities, the flexibility of reactions in everyday life, or one’s performance on new tasks on the behavioral level (Kim et al., 2005; Douglas, 2010a).

### Aphasia and cognitive communication disorders in traumatic brain injury

Studies conducted by Sarno in the 1980s suggested that different types of aphasia are present in about 30% of those affected by TBI (Sarno, 1980; Sarno et al., 1986). Based on their studies in the 1980s, Sarno et al. reported that frank aphasia was present in about 32% of the patients with TBI they studied. However, they pointed out that many of their participants also showed a form of so-called “subclinical aphasia,” which is manifested by problems in word retrieval (anomia) and word fluency (Sarno, 1980). However, the term “subclinical aphasia” has been criticized by researchers (Holland, 1982; Braun and Baribeau, 1987) because this form of communication disorder after TBI tends to result from impaired interaction of cognitive and linguistic abilities. As a result, the terms “Cognitive-Language Disorder” and, more recently, “Cognitive Communication Disorder” have been used to describe communication disorders after TBI that cannot be assessed with conventional aphasia test (Togher et al., 2014a).

Specifically, damage to the fronto-temporal areas can result in aphasic symptoms, such as difficulties in naming and word-finding (Thomsen, 1975; Kerr, 1995), sentence comprehension, or sentence production (Ellis and Peach, 2009; Peach, 2013).

Importantly, language disorders in TBI can be present without focal lesions in the perisylvian language areas of the brain (Demir et al., 2006; Raukola-Lindblom et al., 2021). A much larger percentage of about 80% of individuals with TBI (IwTBI) have disorders in the interactional use of language and at the discourse level. These impairments are subsumed under the term “Cognitive Communication Disorders (CCD)” (Togher et al., 2014b; Christman Buckingham and Sneed, 2020). Impairments of communication after TBI are seen as a consequence of disturbance in the interaction of linguistic and cognitive abilities like planning and controlling of communication-relevant functions (Ardila, 2012; Cannizzaro and Coelho, 2012; MacDonald, 2015). Language impairments in IwTBI are conceptualized as “a window into complex cognitive performance” which is apparent, for example, in gist reasoning, the ability to derive and construct meaning from larger units (Vas et al., 2015). CCD in IwTBI often concerns the pragmatic dimension of language as well as the efficiency and precision of language processing (Channon and Watts, 2003; Bambini and Bara, 2012; Gabbatore et al., 2015; Cummings, 2017; Bosco et al., 2018). For example, even in the absence of anomia, inhibited performance in word fluency tasks under time pressure and other specific constraints may be present in IwTBI (Aschenbrenner et al., 2001; Henry and Crawford, 2004; Whiteside et al., 2016). Studies concerning language in IwTBI find word-finding disorders in more than 70% of individuals (Ponsford et al., 1995) and socio-communicative disorders are mentioned in about one-third of the population of IwTBI (Olver et al., 1996). The discourse level is frequently affected, even in mild forms of TBI (Roth and Hardin, 2021).

### Assessing discourse in traumatic brain injury: Insights into the interplay of language and cognition

The term “discourse” refers to verbal macrostructures, which are realized as either monologues or interactively as dialogues. They can be oral (e.g., everyday narratives, radio messages), written (e.g., text messages, newspaper articles), graphic (e.g., cartoon sequences), or multimodal (e.g., public chats during online conferences). Discourses are more than just a string of sentences. They are characterized by a superordinate structure and the progression of themes across sentence boundaries (van Dijk and Kintsch, 1983.; Zwaan, 1999). Therefore, producing and understanding discourse is regarded as the most complex form of language processing (Cannizzaro and Coelho, 2012; Büttner, 2014). Beyond the sentence level, macrostructural planning processes must be active in order to construct a coherent macrostructure (van Dijk, 1980; Gernsbacher, 1990). To achieve this, skills of selection and sequencing of essential semantic sense units are crucial in addition to the ability to draw inferences to fill causal gaps (Mar, 2004; Kintsch, 2005; Büttner, 2016).

In psycholinguistics, *inference* is described as “the generation of new semantic information from old semantic information in a given context” (Rickheit and Strohner, 1985). Along with the ability to switch perspectives, the ability of inference generation is one of the most important cognitive prerequisites of narration (Büttner, 2016; Zeman, 2016). Processing inferences involves social cognition skills (e.g., Theory of Mind), sufficient working memory capacities, but also EFs (e.g., shifting, updating) (Mar, 2004; Mason and Just, 2011; Singer and Lea, 2012).

In the studies cited here, priority is given to work that focuses on moderate to severe TBIs and discourse skills. Sample sizes ranged from 15 IwTBI (Peach, 2013) to 175 IwTBIs (Ponsford et al., 1995). Time post onset ranged from post-acute (3–6 months p.o., Power et al., 2020) to chronic stage up to 37 years p.o. (Steel et al., 2021). Heterogeneous groups cannot be excluded, for example, Ponsford et al. (1995) states with regard to their sample “175 individuals 2 years post-onset, most of them following severe TBI, but mild to moderate may be present.”

Macrostructural planning processes in individuals with moderate to severe TBI have been examined especially in the context of the coherence of narrative discourses (Lê et al., 2011; Power et al., 2020; Steel et al., 2021). Here, the selection and completeness of essential units, such as key components and obligatory propositions and inferences, can be assessed. For example, sense units that are mentioned by more than 80% of a normative sample are considered to be critical for the thematic progression of the story (Lê et al., 2011). Also, in order to be coherent, utterances must thematically correspond to the topic of a discourse (Cannizzaro and Coelho, 2012). In discourse processing, the interplay of cognitive and linguistic resources comes to light. Discourse processing requires flexible access to cognitive resources (e.g., working memory) as well as intact processes of thematic selection and processes of sequencing. For the selection of essential sense units, or obligatory propositions, different control mechanisms must be active to inhibit irrelevant propositions (e.g., associations). Krueger and Grafman (2008) propose that this goal-directed behavior is guided by so-called *structured event complexes* (SECs), which are “stored” in the prefrontal areas of the brain.

In addition to the severity of the injury and sociodemographic factors (e.g., age, education), discourse impairments in IwTBI can be partly explained by deficits in higher-level cognitive skills (Marini et al., 2014, 2017; Büttner, 2018; Büttner-Kunert et al., 2022). This has been shown by correlations in discourse coherence and scores on tests of cognitive flexibility (Coelho, 2002). Ferstl et al. (2002) demonstrated that working memory impairments negatively influence the ability to draw inferences in narrative texts. In the last few decades, various studies on discourse production in IwTBI have shown that executive control is crucial for the construction of coherent verbal macrostructures (Ylvisaker, 2008; Mozeiko et al., 2011; Ardila, 2012; Lê et al., 2012; Coelho et al., 2013). It is assumed that attention, flexibility, or level of awareness contribute to establishing coherence (Mar, 2004; Zacks and Ferstl, 2016). Also, attentional processes or EFs were recognized as relevant for context-appropriate language processing (Ferstl et al., 2005; Roth and Hardin, 2021).

Various reviews highlight that the consequences of severe TBI can have a negative impact on social and occupational participation as well as on the individual quality of life (Ponsford et al., 2014; Schwenkreis, 2018; Neumann et al., 2019; Falkowska et al., 2021). The massive impact of discourse disorders on social participation has long remained unrecognized, even though individuals directly affected by TBI, as well as their caretakers and relatives, have been highly aware of the negative influence of discourse disorders on their daily lives (Ponsford et al., 2014; Falkowska et al., 2021). Factors influencing participation deficits include impairments in social cognition (Milders, 2019), EFs (Douglas, 2010a), and communication deficits (Douglas et al., 2007a).

Although the relevance of communication and discourse abilities to social participation in TBI has been acknowledged, few assessment procedures have been developed so far (Turkstra et al., 2005; Sohlberg et al., 2019). Classical aphasia test batteries often fail to detect existing discourse deficits in TBI (Cannizzaro and Coelho, 2012; MacDonald, 2017). Especially in the non-English speaking area, there is a lack of diagnostic procedures for the discourse level (Frith et al., 2014; Büttner and Glindemann, 2019; Raukola-Lindblom et al., 2021). Two exceptions to this are the MAKRO-Screening (Büttner, 2018) and the La Trobe Communication Questionnaire (LCQ; Douglas et al., 2000, 2007a). Both use standardized procedures and for both, standard values of a healthy control group as well as evaluation studies (Büttner, 2014, 2016; Büttner-Kunert et al., 2021b,^2022^; Royko and Büttner-Kunert, 2021) or replication studies (Büttner-Kunert et al., 2021a) are available.

The MAKRO-Screening tests verbal macrostructural abilities in discourse production and the comprehension of various types of discourse (narrative, procedural) and the ability to generate inferences in short texts. For the MAKRO-Screening, norms of healthy persons between 18 and 85 years of age (*n* = 172) are available in addition to the education-dependent cut-off score. It is possible to determine the severity of the macrostructural disorder as well as to identify an increased processing time for the respective age range (Büttner, 2018). The LCQ is a multi-perspective procedure that elicits self-assessment as well as the assessment through close others concerning the perceived socio-communicative impairment in TBI. The 30-item questionnaire evaluates conversational behavior in terms of frequency as well as in changes before and after the onset of the injury (Douglas et al., 2007a).

The LCQ is currently the only standardized questionnaire in German-speaking countries that can be used to assess socio-communicative impairment and resources after TBI from the perspective of the affected person (self-assessment) and a close person (Büttner-Kunert et al., 2021b; Falkowska et al., 2021). In evaluation studies on the LCQ, neurologically healthy persons showed a significantly more critical self-assessment (LCQ-S) compared to the assessment of close others (LCQ-O), regardless of sex and educational level (Büttner-Kunert et al., 2021a). Studies on the use of the LCQ in IwTBI have shown that patients tend to rate themselves better than their relatives do (Douglas et al., 2000, 2007a,2007b; Douglas, 2010b). This implies that relatives gave higher scores, meaning greater perceived deficit, in their questionnaires (LCQ-O). Douglas (2010a) outlined a strong pattern between performance on the F-A-S Test (Spreen and Benton, 1969) task and the LCQ. Their study revealed that approximately 30% of the variability in discourse competence was predictable by measuring EFs (e.g., F-A-S, phonemic fluency), although a substantial proportion of variance in the LCQ-ratings was left unexplained.

### Outline of this study

Even though some studies have demonstrated the influence of EFs on the macrostructural organization of discourse, to our knowledge, no study so far has investigated the extent to which discourse production deficits, perceived impairments in socio-communicative participation and verbal executive deficits are interrelated. The goal of our current study was to highlight the impact of cognitive-linguistic deficits (in terms of verbal fluency deficits) on monological as well as dialogical discursive skills.

We assumed that discourse deficits indicate underlying deficits in functions of the executive system. We further suspected that they are manifested in picture-based oral storytelling as well as in conversational behavior and participation in everyday communication.

In our study, we included a word fluency task. Word fluency tasks are of a hybrid nature (Shao et al., 2014). They are frequently used in cognitive science as a measure of executive ability, but also as a measure of lexical access or divergent thinking (Aschenbrenner et al., 2001). We used verbal fluency measures because, on the one hand, they are very widely used in clinical diagnostics and, on the other hand, they are considered a measure of cognitive-linguistic interaction (Whiteside et al., 2016; Rosenkranz et al., 2019). It is precisely because of this interaction that we believe verbal fluency measures are a very good reflection of executive verbal functions.

There is widespread agreement in the research literature that verbal fluency deficits are common after TBI and that these are “probably reflecting executive dysfunction more than linguistic impairment” (Hart et al., 2016).

### Aims and predictions

The aim of our study was to investigate whether and how IwTBI without aphasia perform on discourse processing measures. In this context, we also aimed to analyze the influence of the severity of the initial impairment and verbal fluency impairments on the discourse level. Our research questions were as follows:

(1) Do IwTBI, who do not exhibit aphasic symptoms in the Aachen Aphasia Test (AAT, Huber et al., 1983), show discourse processing impairments, as indicated by their performance on the MAKRO-Screening (subtests *Text production* and *Inferences*) in comparison to non-brain-injured controls?
(2) Do IwTBI show verbal fluency impairments, according to the Regensburg Word Fluency Test (RWT, Aschenbrenner et al., 2001), accompanied by more frequent errors?
(3) Does the discourse performance of IwTBI correlate with their scores on the measures of verbal fluency and task-switching?
(4) How do deficits in the MAKRO-Screening and in executive verbal functions affect the perceived communicative participation? We expected IwTBI to show worse performance when compared to healthy controls. The performance in the subtest *Text production* includes both content analysis (scores indicate the number of obligatory propositions, see Section Discourse Analysis) and qualitative analysis in terms of meta-comments, self-corrections and thematically false utterances. These three categories of utterances are termed “peripheral propositions” because they are not central to the semantic content of the story. Lower scores on the subtest *Text production* indicate difficulties in the narrative production of a coherent and complete story. We postulated that the narrative productions of IwTBI will also differ qualitatively from the controls, namely, that IwTBI will produce more meta-comments and self-corrections, as well as thematically false utterances than those of the control group. Poor performance in the subtest *Inferences* points toward possible impairment in sentence comprehension and inferential reasoning.

Further, we expected IwTBI to produce less items in the semantic condition and the category-switching condition tasks of the RWT. In the presence of possible memory impairments and executive control deficits, we also predicted errors, such as the repetition of aforementioned items and rule violations (e.g., wrong category), to occur more frequently among the patients than in the control group.

We expected to observe a relation between poor performance in the RWT (semantic verbal fluency and task switching) and discourse skills relating to generating inferences and obligatory propositions.

Given the role played by the goal-directed processes involved in the tested macrostructural abilities, we also hypothesized that individuals with lower scores on the MAKRO-Screening will display greater perceived impairment in communicative participation, as indicated by ratings in the two versions of the LCQ.

## Materials and methods

### Participants

A total of 23 IwTBI (4 females/19 males) who met the criteria below were recruited from inpatient rehabilitation facilities in southern Bavaria. On average, around 23 months before testing (range: 7–108 months), participants suffered from severe to moderate TBI, as classified by the Glasgow Coma Scale (GCS) (see Teasdale and Jennett, 1974; range: 3–6 points, mean score: 4). One patient (No. 10, see Table 1) had an initial GCS of 13 but showed a PTA of 18 days, so severe TBI must be considered in this case. The presence of initial aphasia (6 weeks post-onset) was reported in 65% of the sample, no aphasia was reported in 22%, and information on cognitive and language abilities in the initial phase was missing for 13% of participants. PTA and/or duration of PTA was not reported in 47% of the sample.

**TABLE 1:** Background Information of participants with TBI.

Participant	Age	Gender	Education (years)	Time post-onset (months)	GCS	Cause of injury	Initial aphasia	PTA (days)	Main injury (MRT/CT findings)
01	47	M	11	30	3	MVA	n.r.	<14 days	Polytrauma, SDH left, SAH right parietal, hemorrhage falx cerebri, 3 weeks coma
02	40	M	12	16	3	MVA	n.r.	n.r.	Polytrauma, Media infarct (right) with dissection of the A. cerebri interna (right), SAH bifrontal
03	45	W	16	12	3	MVA (hit on bicycle)	Yes	<7 days	DAI
04	31	M	14	8	3	Fall (Table/Homework)	Yes	20 days	Penetrating TBI, diffuse cerebral contusions, multiple hemorrhages left frontal, corpus callosum (splenium), pons (right)
05	20	W	10	37	3	MVA	Yes	28 days	Polytrauma, DAI, petechial microhemorrhages, craniectomy left fronto-parieto-temporal
06	43	M	10	15	3	n.r.	n.r.	Yes, duration n.r.	Polytrauma, Contusion bleeding right-frontal, right-parietal and temporal with subarachnoid spread due to contre-coup-contusion-hemorrhage, calotte fracture
07	58	M	17	14	4	MVA	Yes	20 days	Polytrauma, Contusion bleeding left-frontal, temporo-parietal
08	27	M	13	12	3	Fall (Climbing)	Yes	21 days	Traumatic SAH, epidural hematoma right, traumatic cerebral edema, hemicranioectomy left frontal, DAI
09	34	M	11	13	7	Fall (Work/roof)	Yes	n.r.	n.r.
10	57	M	11	13	13	MVA	Yes	18 days	DAI
11	24	M	11	13	3	Fall (Roof)	Yes	n.r.	Polytrauma, SDH, fronto-temporal hemorrhage
12	30	M	13	20	5	Fall (Climbing)	Yes	16 days	Polytrauma, Cranial calotte fracture, multiple skull base fractures, SDH, SAH and ICH bilateral
13	58	M	11	13	3	MVA	Yes	n.r.	Multiple contusion bleedings
14	25	W	9	37	3	MVA	Yes	Yes, >28 days duration n.r.	Transverse skull base fracture, traumatic SAH, epidural hematoma right, cerebral edema, hemicranioectomy right, additional hypoxic brain injury after reanimation, infarct areas: parieto-occipital, bifrontal left and infratentorial left
15	39	W	11	108	3	MVA	No	<7 days	Right fronto-temporal, left frontal, brainstem
16	34	M	11	14	3	MVA	No	Yes, duration n.r.	Hemicraniectomy left due to epidural hematoma left parietal/brain edema, extensive traumatic SAH right, contusions and small SDH right latero-temporal, DAI
17	39	M	10	7	3	MVA	n.r.	n.r.	Polytrauma, Contusion bleeding temporo-basal
18	42	M	10	18	3	MVA	Yes	14 days	Severe brain contusion, 2 weeks coma
19	31	M	14	20	3	Fall (Homework)	Yes	16 days	Penetrating TBI, diffuse cerebral contusions, multiple hemorrhagic lesions left frontal
20	47	M	11	25	3	n.r.	No	n.r.	SAH right
21	33	M	15	18	3	MVA (electric longboard)	n.r.	39 days	Severe brain contusion, 3 weeks coma
22	41	M	10	60	6	n.r.	Yes	Yes, duration n.r.	n.r.
23	31	M	11	7	6	n.r.	Yes	n.r.	n.r.

GCS, Glasgow Coma Scale; PTA, post-traumatic amnesia; MVA, motor vehicle accident; n.r., not reported; SDH, subdural hematoma; SAH, subarachnoid hemorrhage; DAI, diffuse axonal injury; ICH, Intracerebral hemorrhage. The table provides an overview of the socio-demographic data of the participants and includes medical information, e.g., on the cause and location of the injury.

The participants’ clinical details are presented in Table 1. Participants were recruited from a larger project investigating discourse and cognitive impairments in individuals with neuropragmatic disorders. For this study, we included participants with a history of TBI in the post-acute and chronic phase (at least 6 months post-injury). Additionally, participants were required to meet the following criteria:

• no aphasic symptoms on the sentence level at the time of examination, as assessed by neuropsychological or speech therapists during rehabilitation (f. e., percentile rank > 95 in the subtests *Token Test*, *Naming*, and *Written Language* of the AAT; Huber et al., 1983),
• a German language background,
• aged between 18 and 60 years at the time of injury,
• sufficient hearing ability and eyesight,
• no history of previous neurologic or psychiatric diseases (e.g., schizophrenia), substance abuse, or developmental delay,
• no severe attention or memory disorders,
• no visual perception deficits and
• no dysarthria.

As part of the aforementioned larger project, data from 23 healthy individuals were also taken into account. All participants were right-handed.

The control group was comparable to the experimental group regarding age (mean: 36.87; *SD*: 12.57), gender (4 female/19 males), and level of education (mean: 13.26; *SD*: 1.82). The groups did not differ in age (*T* [44] = 0.354, *p* = 0.068) or years of education (*T* [44] = −2.011, *p* = 0.269) (see Table 2). Cognitive scores of the MoCA (*Montreal Cognitive Assessment*; Nasreddine et al., 2005) ranged from 16 to 28 (mean = 23.61, SD = 2.872) for the group with TBI (see Table 3).

**TABLE 2:** Demographic information for all participants.

	TBI (*n* = 23)	Control (*n* = 23)
**Age**		
Mean (SD)	38.09 (10.67)	36,87 (12.88)
Median	39	36
Range	20–58	21–56
**Education**		
Mean (SD)	12.04 (2.26)	12.80 (2.01)
Median	11	12
Range	9–17	10–16

Details on age and education (in years) of the participants with TBI and the control group are summarized.

**TABLE 3:** Scores for the MoCA (subtests and total score) for IwTBI (*n* = 23).

Participant	Visuospatial/executive*^a^* (max. 5)	Naming (max. 3)	Attention*^b^* (max. 6)	Language*^c^* (max. 3)	Abstraction (max. 2)	Delayed recall (max. 5)	Orientation (max. 6)	Sum all subtests (max. 30)	1 point for educatio*n* ≤ 12*^d^*	Total score
01	4	3	4	3	0	0	6	20	Yes	21
02	4	3	6	2	2	3	6	26	Yes	27
03	4	3	3	0	0	0	6	16	No	16
04	5	3	6	1	2	0	5	22	No	22
05	4	2	6	1	2	2	6	23	Yes	24
06	3	3	5	1	2	0	5	19	Yes	20
07	4	3	5	2	2	2	4	22	No	22
08	5	3	5	2	2	3	6	26	No	26
09	4	3	4	2	2	4	5	24	Yes	25
10	3	3	3	1	2	3	5	20	Yes	21
11	3	3	3	1	2	3	4	19	Yes	20
12	3	3	4	2	2	4	5	23	No	23
13	5	3	5	2	2	4	5	26	Yes	27
14	4	3	6	3	2	0	5	23	Yes	24
15	5	3	6	2	2	2	6	26	Yes	27
16	3	3	5	2	2	3	5	23	Yes	24
17	5	3	5	1	2	2	6	24	Yes	25
18	5	3	5	0	2	2	6	23	Yes	24
19	5	3	6	3	1	3	6	27	No	27
20	5	3	5	2	2	4	6	27	Yes	28
21	5	3	5	2	2	0	5	22	No	22
22	n.r.*^e^*	n.r.	n.r.	n.r.	n.r.	n.r.	n.r.	n.r.	n.r.	24
23	n.r.	n.r.	n.r.	n.r.	n.r.	n.r.	n.r.	n.r.	n.r.	24

^a^Visuospatial/executive = sum of the subtests: trail making test, cube copying, clock drawing.

^b^Attention = sum of the subtests: list of digits, list of letters, serial subtraction.

^c^Language = sum of the subtests: sentence repetition, word fluency.

^d^One additional point was added for participants with years of education ≤12.

^e^n.r. = not reported; no data for detailed score listing is available for participant 22 and participant 23.

The project investigating communicative and cognitive impairments in neuropragmatic disorders received ethical approval from the German Linguistic Society (DGFS 2016-13-170208). All patients participated voluntarily, were informed about the study in written and spoken word, and gave their written consent.

### Materials

*MAKRO Screening (Büttner, 2018).* To evaluate discourse production abilities, two subtests of the MAKRO Screening were conducted: *Text production* and *Inferences*. In the subtest *Text production*, participants were asked to sort several pictures into a coherent sequence and to tell a story based on the pictures with the instructions: “*Please tell a story based on these pictures. Look closely at every picture. Tell the story as though to someone who cannot see the pictures. Please also mention what may have happened in between the frames.*” While sorting the pictures, assistance was provided, if necessary. The pictures remained in front of the participants while they told the story. There was no time limit for the narrative task, so participants could end the story at their own pace. The task was conducted with two picture stories, which differed in length and complexity (either four or eight pictures). Narratives were evaluated regarding story completeness. Each obligatory proposition or inference was awarded with one point (max. 12 points in the shorter story, max. 18 points in the longer story).

Besides the content analysis, qualitative characteristics of text production were also examined. In this context, the transcripts were analyzed with regard to meta-comments, self-correction and thematically false units (see Appendix for examples).

In the subtest *Inferences*, participants were instructed to read a short text with a sentence gap and to produce one to two sentences that would provide the missing link for the given outcome. The challenge in the task is to draw a logical conclusion as to which information is missing, also known as *bridging inference*. The instructions given were: “*Please complete the missing line. What might have happened in between? Write or say a sentence that explains the outcome of the story.*” The participants were free to give either a written or an oral answer. For each correct response, a maximum of three points could be awarded (max. 30 points overall). Two points were assigned for answers with linguistic-formal errors. Thematically related key terms or ellipses were graded with one point. No points were assigned for confabulations, perseverations, or omissions (see Appendix for examples).

*Regensburg Word Fluency Test* (RWT; Aschenbrenner et al., 2001). As a verbal fluency measure, the RWT was conducted for two conditions: a semantic and a category-switching condition. In the semantic condition (*RWT-sem*), participants were asked to name as many words from a semantic category as possible in the time span of two minutes (“food”). In the category-switching condition (*RWT- switch*), patients shifted between two categories (e.g., “sports” and “fruit”). For the RWT, percentile ranks are available for the total number of words produced as well as the error types for example repetitions, rule violations (e.g., wrong semantic category).

*Montreal Cognitive Assessment* (MoCA; Nasreddine et al., 2005). The MoCA is a quick tool used to detect mild cognitive impairment (MCI) and dementia symptoms. It was originally developed to assess global cognitive functions in patients with Alzheimer’s disease and was subsequently validated in various clinical settings. The MoCA is reported to be a valid tool for detecting subtle cognitive impairment in mild TBI and differentiates between cognitive functioning in mild to severe TBI, although it should not replace an extensive neuropsychological assessment (Mishra et al., 2020). The test allows for a short screening of various cognitive functions like short-term memory, attention, and working memory (e.g., forward and backward digit span), visuospatial abilities (e.g., clock drawing test), EFs (e.g., alternating trail-making test), and orientation to time and place. For the MoCA, both norm data and cut-off scores are available.^1^

*La Trobe Communication Questionnaire* (LCQ; Douglas et al., 2000). The LCQ was developed as a tool to assess perceived communicative ability between young adults following TBI and their relative or caretaker. The questionnaire consists of 30 items that are to be rated on a Likert-scale referring to the frequency of the occurrence of a specific communicative behavior (from 1 = *never or rarely* to 4 = *usually or always*). In the self-assessment version (LCQ-S), participants filled out the questionnaire referring to their own perceived communicative behavior. In the relative’s report (LCQ-O), significant others evaluated the patient’s communicative behavior. The parallel versions may indicate discrepancies between the patient’s self-awareness and the relative’s perception of the efficiency in everyday communication. The assessment was either conducted orally or in written form.

### Procedure

Participants were invited for individual testing sessions in a quiet clinical room, either in the rehabilitation institution (for the patients) or the university’s rooms (for the control group). Participants completed the assessment within two hours, which were divided into two sessions conducted on the same day.

The test sessions were recorded on video and audio and the scoring procedure was performed offline. An additional 20% of the story narratives (subtest Inferences and subtest Text production) were analyzed by a second rater. Inter-rater reliability (IRR) was considered as “acceptable” if the IRR score was at least 75% (e.g., Cohen, 1988). The IRR score for the subtest Text production was 82% and for the subtest Inferences 87% indicating an acceptable IRR. The order of the cognitive and discourse tasks was randomized.

### Discourse analysis

The analysis was carried out by two authors (SB, ZF). Each transcript was controlled by at least one other investigator (JBK). Deviations were discussed.

Audio samples from the narratives (*Text production*, short and long story) were transcribed orthographically. Non-word utterances, filled pauses and other dysfluencies were ignored in this analysis, as we focused on the semantic-lexical content.. Most transcripts were presented as monologues from the participants. The investigator only intervened if the participant asked for assistance on the task. Utterances from the investigator were excluded from further analysis.

After transcription, a content-related analysis was conducted. As part of the MAKRO scoring, transcripts were rated according to whether all critical events in the stories were mentioned. So-called *obligatory propositions* were identified, that is, content elements that were produced by more than 80% of participants in the norm sample of the MAKRO and were critical for the thematic progression of the story (Büttner, 2018).

In addition to the critical elements of the story, thematically false utterances, meta-comments, and self-corrections were identified. These three categories of utterances are termed “peripheral propositions.” Narrative elements unrelated to the content of the story or conclusions that seemed inappropriate considering the given pictures were referred to as thematically false utterances. Utterances that distracted from the narrative situation (e.g., commenting on the design of the pictures, personal comments such as “I guess” or “I don’t know why”) were classified as meta-comments. Syntactically incomplete sentences that were rephrased subsequently were counted as self-corrections.

### Data analysis

All data were entered into IBM Corp Statistics 26.0. Variance homogeneity was assessed by the Levene Test and confirmed for the raw scores and errors of the RWT as well as for both LCQ versions. For between-group measures, unpaired *t*-tests were applied. For inadequate variance homogeneity, non-parametric tests (Mann—Whitney *U* test) were applied. According to the directionality of our hypotheses, seeing as we expected a worse performance from the TBI group, all tests were one-sided. A significance level of *p* < 0.05 was set as an appropriate level for all analyses in this study. Standard multiple regression analysis was used to evaluate the variance (R^2^ and adjusted R^2^) in perceived communicative participation (LCQ total score) accounted for by verbal executive function (RWT) and discourse production impairment (MAKRO “Text Production” (total score) and amount of peripheral propositions), all entered individually). To determine Type 1 errors arising from multiple comparisons, Holm’s sequential procedure was applied when necessary. Holm’s correction for Type I error is regarded as effective as the traditional Bonferroni method while retaining more statistical power (Eichstaedt et al., 2013). To test for correlation between discourse processing variables and verbal fluency measures, we calculated the Pearson correlation coefficient (*r*).

## Results

(1) Do IwTBI, who do not exhibit aphasic symptoms in the Aachen Aphasia Test (AAT, Huber et al., 1983), show discourse processing impairments, as indicated by their performance on the MAKRO-Screening (subtests Text production and Inferences) in comparison to non-brain-injured controls?

The comparison between the *Text production* scores of the two groups revealed that participants with TBI had lower scores (mean = 22.4*3*, *SD* = 4.28) than the controls (mean = 27.96, *SD* = 1.29) and that this difference is significant (Mann–Whitney *U* test, *U* = 470, *z* = −4.56, *p* < 0.001). Furthermore, we compared the discourse performance of the two groups in terms of the occurrence of meta-comments, self-corrections and thematically false utterances. Transcripts were segmented in words and utterances, and a qualitative assessment was conducted (see Table 4 for details). Thematically false utterances only occurred in the TBI group, but not in the control group. The both groups showed no significant difference in self-corrections (*U* = 163, *z* = −0.81, *p* = 0.22). The amount of meta-comments was significantly higher in the TBI group (*U* = 120, *z* = −2.32, *p* = 0.015).

**TABLE 4:** Overall results in the MAKRO subtests.

Variables	TBI (*n* = 23)				Controls (*n* = 23)			
		
	Min.	Max.	Mean	SD	Min.	Max.	Mean	SD
MAKRO: Text production (score)	13	29	22.43	4.28	25	30	27.96	1.26
Meta-Comments	0	22	3.30	5.15	0	3	0.70	1.08
Self-corrections	0	10	1.85	2.70	0	5	1.15	1.57
Thematic-false units	0	2	0.30	0.65	0	0	0	0
MAKRO: Inferences (score)	9	30	23.83	6.18	28	30	29.61	0.491

Results of the subtests Text production (score and qualitative analysis) and Inferences (score) are listed (range, mean, standard deviation).

**FIGURE 1:**
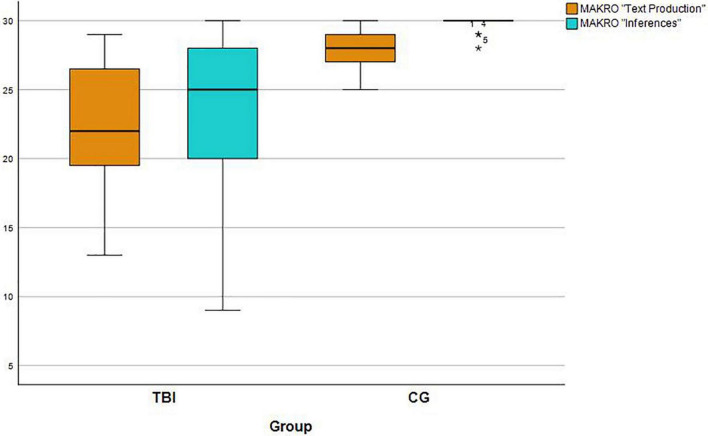
Group performance (IwTBI and Control Group) in the MAKRO subtests “Text Production” and “Inferences.” The maximum score per subtest is 30 points.

In the subtest MAKRO Inferences IwTBI had lower scores than the controls, too. The groups differed significantly (*U* = 462, *z* = −4.73, *p* < 0.001). While the mean score of the IwTBI was 23.83 (*SD* = 6.18), the controls reached a mean score of 29.61 (*SD* = 0.49; see Table 4). See Figure 1 for the comparison of the two groups in these subtests. Ceiling effects have emerged for the control group in both *Text production* and *Inferences*, as there is a maximum point value of 30.

(2) Do IwTBI show verbal fluency impairments, according to the RWT, accompanied by more frequent errors?

**FIGURE 2:**
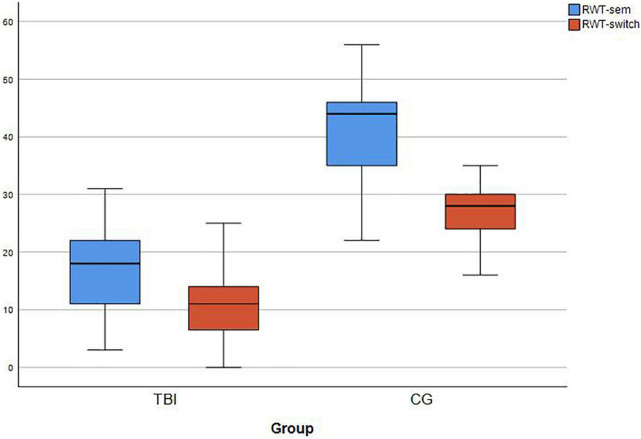
Boxplots for RWT raw scores for IwTBI and the Control Group on the two conditions of the verbal fluency task. RWT-sem: semantic fluency for “food”; RWT-switch: switching category “sports and fruits.” Each task was performed for 2 min.

First, the raw scores of the semantic condition differed significantly (*T* [44] = −8.91, *p* < 0.001). The mean scores of IwTBI were clearly lower (*mean* = 17.52 (*SD* = 7.86) than those of the control group (*mean* = 41.70, *SD* = 10.35). For the category-switching condition, similar results can be reported (TBI: *mean* = 10.91, *SD* = 6.37; Controls: *mean* = 26.74, *SD* = 5.06, see Figure 2). Once again, the *t*-test showed significant differences (*T* [44] = −9.32, *p* < 0.001). Upon further analysis, the error types of the RWT-sem and RWT-switch were summarized. There a statistically significant difference was found for rule violations between IwTBI and the control group (*U* = 131, *z* = −3.628, *p* < 0.001) as well as for repetition errors (*U* = 142,50, *z* = −3.162, *p* < 0.001; see Table 5).

**TABLE 5:** Descriptive statistics for performance and errors in the verbal fluency tasks.

	TBI (*n* = 23)				Control (*n* = 23)			
		
Variables	Min.	Max.	Mean	SD	Min.	Max.	Mean	SD
**Semantic condition**								
Items (raw score)	3	31	17.52	7.86	22	56	41.70	10.35
Percentiles	0	35	4.09	7.83	35	98	70.09	19.21
Repetitions	0	3	0.83	0.89	0	2	0.22	0.66
Rule violations	0	2	0.18	0.50	0	0	0	0
**Category switching**								
Items (raw score)	0	25	10.91	6.37	16	35	26.74	5.06
Percentiles	0	69	6.09	15.90	36	98	70.48	21.90
Repetitions	0	2	0.57	0.79	0	0	0	0
Rule violations	0	5	0.91	1.28	0	3	0.13	0.62

Results of the verbal fluency measures are given here. Raw scores and percentiles of items are listed as well as errors for each of the subtests.

(3) Does the discourse performance of the IwTBI correlate with scores on the measures of verbal fluency and task-switching?

Prior to evaluating associations between discourse production, communicative participation, and measures of executive function, correlations with demographic and injury-related variables were calculated. For controlling the family-wise error rate Holm’s-procedure was applied to *p*-values. Therefore, only *p*-values <0.5 are reported. None of the correlations between the subtests of the MAKRO, LCQ-S, LCQ-O, time post-injury and age reached significance. There was a significant and moderate correlation (*r* = 0.450, *p* = 0.048) between the severity of the injury (GCS) and the subtest *Inferences*.

To determine a possible influence of mild cognitive impairment on the MAKRO subtests and the LCQ, correlations between the discourse tests and the MoCA were performed. None of the discourse tasks showed significant correlations with the cognitive screening.

To assess the potential relationship between discourse performance and executive functioning for IwTBI, Pearson product-moment correlations were calculated for the RWT scores, the MAKRO subtests, and the LCQ scores (see Table 6). First, we conducted the correlations for the oral discourse assessment (MAKRO Text Production) and the verbal fluency measures. There was no correlation between the scores of the MAKRO subtest *Text production*, which measures story completeness, and the RWT tasks. Second, we put the focus on correlations for verbal fluency measures and the LCQ. Scores of the LCQ-S correlated moderately with those of the RWT-sem (0.410, *p* < 0.05) and the RWT-switch (0.472, *p* < 0.05). The evaluation of close-others showed no significant correlation with the conducted measures of verbal fluency. In the next step, we conducted the correlations for the Subtest Inferences and the verbal fluency measures.

**TABLE 6:** Correlations between measures of executive functions and discourse performance for IwTBI (*N* = 23).

	Text production	Inferences	RWT sem	RWT switch	LCQ-S	LCQ-O
Text Production	−	–0.210	0.058	–0.097	0.196	0.237
Inferences	–0.210	−	0.604*	0.610*	0.060	–0.045
RWT-sem	0.058	0604*	−	0.859*	0.410*	0.197
RWT-switch	–0.097	0.610	0.859*	−	0.472*	0.147
LCQ-S	0.196	0.060	0.410*	0.472*	−	0.493*
LCQ-O	0.237	–0.045	0.197	0.147	0.493*	−

The table reports the Pearson’s R values for the MAKRO subtests Text Production and Inferences, the scores of the verbal fluency tasks RWT-sem and RWT-switch, the LCQ total scores of self-assessment (LCQ-S) and assessment of close others (LCQ-O). Bold values represent significant correlation. Asterisks (*) denote correlations *p* < 0.05 after multiple comparison correction (Holm’s procedure).

Scores of the subtest *Inferences* correlated strongly with the RWT-sem (*r* = 0.604, *p* < 0.05) and with the RWT-switch (*r* = 0.610, *p* < 0.05. We further conducted a partial correlation to measure the association between the subtest *Inferences* and the RWT tasks while controlling the effect of the severity of injury. Even though this adjustment was carried out, moderate and significant correlations were still observed for the RWT-sem (0.427, *p* < 0.05) as well as for the RWT-switch (*r* = 0.437).

(4) Do people with impairments in the MAKRO-Screening display problems in perceived communicative participation?

**FIGURE 3:**
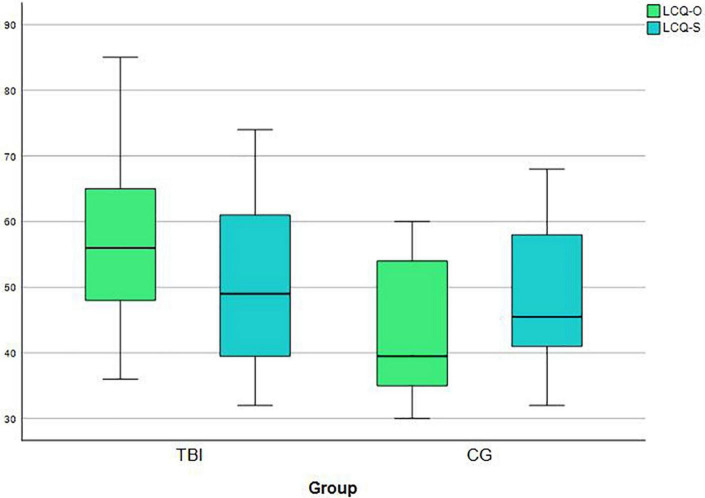
Boxplots for LCQ scores for IwTBI and the Control Group in the self-assessment (LCQ-S) and in the evaluation of close-others (LCQ-O). Higher scores are reflecting greater perceived difficulties in communicative participation. Possible values are between 30 to 120 points.

Our fourth research question focused on the difference between IwTBI and the control group in communicative participation. Levene’s Test of Equality of Variances showed homogeneity of variance for the LCQ-O (*T* [44] = 4.34, *p* = 0.212) and the LCQ-S (*T* [44] = −0.09, *p* = 0.216). Significant differences between the two groups were shown by *t*-tests for independent samples in the LCQ-O (*T* [44] = 4.337, *p* < 0.001), but not in the self-assessment (*T* [44] = 0.570, *p* = 0.569). Relatives of IwTBI reported significantly higher total scores than relatives of the control group, which implies greater perceived impairments in communicative participation (see Figure 3).

There was also a significant correlation between the LCQ-S and the LCQ-O (*r* = 0.493, *p* = 0.017; see Table 6) for IwTBI. The correlation between the LCQ-S and the LCQ-O was slightly stronger in the control group (*r* = 0.784, *p* < 0.001).

We were further interested in the influence of macrostructural variables and EFs on perceived impairments in communicative participation. Thus, the groups (IwTBI and controls) were separated by their performance on the subtest *Text production* using a cut-off score of 26 points (2 SD lower than the mean value of the normative sample) (Büttner, 2018). The first group’s performance (score > 26 points) in storytelling was comparable to the normative sample. The second group (score ≤ 26 points) displayed significantly inferior abilities in storytelling.

A more in-depth analysis was conducted using a series of Mann–Whitney *U* tests to compare impaired storytelling with perceived communicative participation and with verbal EFs. Results showed statistically significant differences between the two groups in the LCQ assessment of close-others (LCQ-O, *U* = 157, *z* = −2.284, *p* = 0.010), but not for the self-assessment (LCQ-S, *U* = 259, *z* = 0.206, *p* = 0.418. There was a statistically significant difference in the LCQ-O between Group 1 (*M*Rank = 19.54) and Group 2 (*M*Rank = 28.65), indicating that the group with poor storytelling skills showed greater perceived communicative impairment as evaluated by their close-others (e.g., relatives, friends, or care-takers). Group 2 had significantly reduced semantic fluency (RWT-sem, *U* = 401.00, *z* = 3.127, *p* = 0.002) and poorer task-switching abilities (RWT-switch, *U* = 396.00, *z* = 3.016, *p* = 0.003). Group 2 displayed a lower mean rank on the MoCA (*U* = 409.50, *z* = 3.464 *p* < 0.001). See Table 7 details.

**TABLE 7:** Results of the Mann–Whitney-*U*-Tests for group comparisons, MAKRO subtest “Text Production” (*n* = 46).

	MAKRO cut-off TP	*N*	*M*Rank	Mann–Whitney-*U*	Wilcox-W
PR RWT-sem	Group 1	26	29.77	423.00	774.00
	Group 2	20	15.35		
PR RWT-switch	Group 1	26	29.21	408.50	759.50
	Group 2	20	16.08		
LCQ-O	Group 1	26	20.65	157.00	508.00
	Group 2	20	28.14		
LCQ-S	Group 1	26	25.08	259.00	584.00
	Group 2	20	23.82		
MOCA	Group 1	26	19.18	409.50	760.50
	Group 2	20	15.39		

The table provides an overview of ranks in term of verbal fluency performance (percentile rank), perceived communicative participation and overall cognitive status, grouped depending on text production performance. Group 1: up to and over Cut-off: ≥27; Group 2: under Cut-off: <27.

The effect size according to Cohen (1988) was calculated. We used the correlation coefficient, Pearson’s *r*, which is typically applied to characterize the degree to which one variable can be predicted by another (Funder and Ozer, 2019). With reference to Funder and Ozer (2019) we expected *r* = 0.05 to correspond to a very small effect, *r* = 0.10 to a small effect, *r* = 0.20 to a medium effect, *r* = 0.30 to a large effect, and *r* ≥ 0.40 to a very large effect. The effect size was large (*r* = 0.35) for the evaluation of perceived communicative impairments (LCQ-O). For verbal fluency, the effect size was very large for the RWT-sem (*r* = 0.44) and for task-switching (*r* = 0.43) as well.

To assess the contribution of discourse performance and verbal fluency to communicative participation for the whole sample, standard regression analysis was performed after screening for outliers in adherence to the assumptions of multivariate analyses. Regression analyses were used to examine the relation between LCQ-O (dependent variable, DV) and RWT-sem, total score of peripheral propositions (self-corrections, thematic-false utterances, meta-comments) and the score in the subtest *Text production*. Table 8 shows bivariate correlations between the variables included in the regression equation as well as unstandardized (*B*) and standardized (*b*) regression coefficients, the semipartial correlations (*sr*^2^), *R*, *R*^2^, and adjusted *R*^2^.

**TABLE 8:** Standard multiple regression of discourse production variables and RWT-sem on LCQ-O total scores for IwTBI and controls (*n* = 46).

Variable	LCQ-O (DV)	RWT sem	Text production	Peripheral propositions	β	*B*	sr^2^
RWT sem	–0.641		0.576	–0.311	–0.606	–0.238	–0.196
Text production	–0.331	0.576		–0.095	0125	0.332	
Peripheral propositions	0.314	–0.331	–0.095		0.006	0.021	
M	50.18	38.17	25.28	3.67			
SD	14.381	36.635	4.188	5.398			
*R* ^2^					0.425		
Adj. *R*					0.378		
*R*					0.652		

The model has no auto-correlation, as the value of the Durbin-Watson statistic is 1.876. RWT-sem, *Text production* and peripheral propositions were able to statistically significantly predict perceived communicative participation, *F* (3, 39) = 8.886, *p* < 0.001.

The *R*^2^ for the overall model was 0.421 (adjusted *R*^2^ = 0.378), indicative of a high goodness-of-fit according to Cohen (1988). Only the RWT-sem score yielded a regression coefficient that deviated significantly from 0 (*sr*^2^ = −0.196, *p* < 0.001), indicating that 19.62% of the variance in LCQ-O total scores was attributable to RWT-sem as a unique factor.

The *Text production* score and the total score of peripheral propositions did not make significantly unique contributions to the prediction of variability in the total score of the LCQ-O.

## Discussion

In this study, we sought to identify impairments in discourse performance in IwTBI and its interaction with verbal executive dysfunctions and communicative participation. For this reason we selected two discourse tasks from the MAKRO-Screening, *Text Production* and *Inferences*, to assess macrostructural abilities in different conditions. Furthermore, we were interested in the impact of impaired verbal fluency measures in different conditions on discourse performance and perceived deficits in communicative participation from different perspectives.

The first research question focused on the measures of discourse production and inferential reasoning. Consistent with previous studies assessing narrative discourse processing (Lê et al., 2011; Coelho et al., 2013; Marini et al., 2017; Büttner-Kunert et al., 2022) we found lower scores in participants with TBI than in the control group. As the score of *Text Production* reflects the number of obligatory propositions, IwTBI had obvious difficulties in finding all the propositions necessary for telling the story in a coherent and comprehensive way. This goes in line with the findings of Mozeiko et al. (2011) who found lower scores in story completeness in a large sample of 171 Individuals with moderate to severe TBI in comparison to controls. The results of our study indicate that the measure of completeness, which refers to the amount of critical components of a story, is sensitive in detecting discourse impairments in IwTBI.

Moreover, a worse performance in the subtest *Inferences* could be confirmed. This result points to impairments in text comprehension and inferential reasoning. IwTBI displayed significantly greater difficulties in generating bridging inferences to fill the causal gaps in the short texts presented. Our findings corroborate the results of Ferstl et al. (2002) who mention impaired inference generation in TBI, especially after left prefrontal lesions.

We also investigated qualitative differences in the narrative productions for of TBI and control participants. Consistent with our predictions, we found the produced stories of IwTBI more frequently disrupted by peripheral propositions (self-corrections, thematic false utterances, meta-commentaries). The more frequent occurrence of peripheral propositions in the TBI group can be interpreted in terms of planning deficits while constructing a coherent narrative. They also point toward efforts of monitoring, meaning that participants seemed to be aware of misconstructions and tried to adapt planning processes online. Still, the frequent occurrence of self-corrections may indicate that monitoring efforts are delayed and can not engage on time. As to whether self-corrections may suggest syntactic planning deficits or semantic-lexical retrieval difficulties cannot be definitively answered based on our data. In respect to performance in the verbal fluency tasks, a semantic retrieval deficit in TBI can not be ruled out. In contrast, for example, Ellis and Peach (2009) suspected a syntactic planning deficit in IwTBI. To answer this question, further investigations should be conducted. The more frequent occurrence of meta-comments is in line with previous results (Büttner-Kunert et al., 2022). The production of meta-comments can be explained in terms of the inability to inhibit irrelevant content leading outside the narrative frame. These problems highlight difficulties in establishing a coherent situational model, which requires sufficient planning skills. It seems that IwTBI display more problems in keeping attention on the story frame and suppressing irrelevant contents.

Another qualitative measure was the amount of thematically false units. Contrary to our expectations, only few thematic-false utterances appeared in the TBI group. This is surprising considering the relevant group differences we found in the *Inferences* test, where the ability to draw conclusions was also tested. Also, our results diverge from previous results (Falkowska and Büttner-Kunert, 2020; Büttner-Kunert et al., 2022), where a higher percentage of thematically false units was reported for the TBI group compared to our results. In this earlier study, the difference in peripheral utterances was more prevalent between the groups. These inconsistent results may be explained by other factors such as the characteristics of post-acute and chronic stages, as the groups differed in regard to time post-onset. For example, the occurrence of thematically false content in narratives could be a characteristic of an earlier stage of injury. Follow-up studies by Power et al. (2020) and Snow et al. (1999) showed that fewer coherence errors occur with an increase in time post onset. Significant improvement over time in discourse production could be associated with an improvement in monitoring skills achieved by behavioral (e.g., GIST Training, Hawley and Newman, 2010) or cognitive-linguistic therapy (Pragmatic Training, Gabbatore et al., 2015). However, the occurrence of thematically false utterances should still be considered as a distinguishing criterion between the populations, as it seems highly uncommon in the healthy population, consistent with the previous analyses by Büttner-Kunert et al. (2022).

Our second research question concerned the performance of IwTBI in two verbal fluency tasks. We expected IwTBI to display verbal fluency impairments and more errors in the tasks. Our expectations were confirmed by a lower score in both the semantic and category-switching condition. Group differences were significant in both cases. The control group produced more than double the number of items during the tasks than IwTBI. Accordingly, percentile scores were below-average for the TBI group. Significant group differences for the amount of rule violations were shown. We found more frequent rule violations like items from the wrong semantic category or perseverations in the TBI group than in the control group.

Various studies highlight that IwTB have long-lasting cognitive-linguistic difficulties, frequently resulting in a breakdown of interpersonal communication in various everyday situations (Douglas et al., 2019). The studies of Douglas et al. (2015), but also of Struchen et al. (2008), were able to demonstrate the usefulness of the LCQ for the assessment of communicative participation deficits in IwTBI. The present work is the first to implement the German version of the LCQ with a cohort of IwTBI to not only demonstrate the extent of socio-communicative participation, but also to examine discrepancies between self-assessment and the assessment of close-others.

Our results showed that IwTBI differed significantly from control subjects, but only in the assessment of close-others. The fact that IwTBI rate themselves significantly better than their relatives could be due to a lack of self-awareness and coherence monitoring.

In our work, we only compared the total scores of the questionnaires and thus did not exhaust the full possibilities offered by the LCQ. A finer analysis of impaired factors (e.g., “Conversational Fluency” or “Task Management”) or individual items could provide even more clarification on the influence of impaired verbal EFs on conversational competence (Douglas et al., 2007a; Struchen et al., 2008; Douglas, 2010a).

Not only was our study the first to demonstrate the implementation of the German version of the LCQ, we also found a significant impact of verbal fluency performance on communicative self -perception. In our study, the percentage of variability was lower than that which was found by Douglas (2010a). One reason for this could be that in the current study, the focus was on the examination of the perspective of close-others, whereas Douglas (2010a) focused on self-perception. Based on our findings, it could be assumed that a disturbance of cognitive-linguistic control mechanisms, which are required for verbal fluency performance, discloses itself also in the perception of close-others. Reduced verbal fluency performances also refer to a diminished ability of idea generation. Therefore, this could also be reflected in the perception of the relatives and less in the self-assessment, especially if self-awareness and monitoring (e.g., of thematic progression in story telling or everyday conversation) are impaired.

In addition, we were able to demonstrate that individuals with lower scores on the subtest *Text production* also displayed higher scores in the LCQ-O. Higher scores in the LCQ indicate greater perceived frequency of impairments in communication. This is an interesting finding, which demonstrates the conceptual relationship between the relevance of verbal planning in narrative texts and in conversational behavior.

The findings with reference to the fluency task imply that an undisturbed access to semantic representations is significantly involved in the generation of bridging inferences. The significant correlations between the subtest Inferences and the verbal fluency task refer to similar processes that create meaning. Both tasks require verbal planning, which encompasses flexible and fast access to semantic representations. Furthermore, our results reinforce the assumption that “executive functions and macrostructural linguistic abilities are orchestrating the narrative process” (Büttner, 2016).

### Limitations of the study

We tried to indicate the severity of TBI by means of the GCS, but the presence of posttraumatic amnesia (PTA) should also be taken into account for the classification of severity and for predicting the individual outcome. We were unfortunately unable to address this in detail because clinical data on this were insufficient in about half of the subjects.

Although we analyzed the influence of some executive functions such as task-switching and verbal fluency, there are other cognitive parameters (e.g., attention, planning skills, working memory), that we did tested only via screening (MoCA). Further research is necessary to investigate the influence of these cognitive functions on factors like discourse production. Highly interesting “candidates” for this are social cognition and abstract thinking. Future studies should look more closely at the impact of reduced Theory of Mind or empathy on discourse competence (Stronach and Turkstra, 2008; Bosco et al., 2018; Allain et al., 2019) or gist reasoning (Vas et al., 2015).

### Clinical implications

Overall, our results indicate the high relevance of discourse assessments in patients with TBI. Even though the participants had no aphasic symptoms on the sentence level at the time of examination, we detected significant impairments in telling the stories in a coherent and comprehensive way. Furthermore, patients showed difficulties in their perception of the deficits. Therefore, in order to diagnose, treat, and identify the functional impact, the collaboration between neuropsychologists and speech-language-therapists should be aimed for. This includes the development of interdisciplinary guidelines for the rehabilitation of people with TBI in all countries and languages as well as coaching concepts for the adequate and supportive management of discourse disorders for relatives of the affected persons, caregivers, but also for public service employees (Togher et al., 2010, 2016).

## Data availability statement

The raw data supporting the conclusions of this article will be made available by the authors, without undue reservation.

## Ethics statement

The studies involving human participants were reviewed and approved by Ethikkommission der Deutschen Gesellschaft für Sprachwissenschaft (DGfS). The patients/participants provided their written informed consent to participate in this study.

## Author contributions

JB-K contributed to funding and conception and design of the study. JB-K, SB, and ZF organized the database, performed the statistical analysis, and wrote sections of the manuscript. CO and TR supported the data collection. All authors contributed to the article and approved the submitted version.

## Footnotes

1
https://www.mocatest.org/the-moca-test/

## Appendix

Examples of the subtests *Text Production* and *Inferences* of the MAKRO Screening are described in the following (translated from German). A detailed test description and the cartoon sequences can be found in the MAKRO manual (https://www.nat-verlag.de/programm/diagnostik/makro/). If interested, the materials can be requested directly from the author (JBK) for personal use.

### Subtest text production

The shorter story (“The Dinner”) consists of 4 pictures and of 12 obligatory propositions and inferences. The longer story consists of 8 pictures. It has 18 obligatory propositions and inferences und is made up by more episodes than the shorter one.

Instruction: “*Please tell a story related to the cartoons. Please look at the pictures carefully.*

*Tell the story in such a way that even someone who doesn’t know the cartoon understands the story. Also tell what happens between the pictures. Please don’t just describe the pictures, tell the story!”*

Example (shorter story)

Plot of “The Dinner”

The pictures show a situation at dinner. The husband (person 1) is sitting at the table and is reading his newspaper. Under the table, the family’s cat is sitting in a basket. The wife (person 2) is serving a fish, and then she goes back into the kitchen. In the meantime, the cat steals the fish. The man (person 1) does not notice [INFERENCE 1]. The woman (person 2) comes back and yells at the man. She thinks that her husband did not wait for her with having dinner [INFERENCE 2]. The cat is full and satisfied and licks his paw.

**Table:**

^•^ P1: be there (man/person 1)	^•^ P8: come back (woman/person 2)
^•^P2: be there (woman/person 2)	^•^P9: be away (food)
^•^ P3: be there (cat)	^•^ P10: see (woman, P9)
^•^ P4: bring (woman, food)	^•^ P11: suspect (person 2, person 1)
^•^ P5: read (man, newspaper)	^•^ P12: be clueless (man)
^•^ P6: notice (cat, fish)	^•^ P13: react (person 2, to person 1, negatively)
^•^ P7: get (cat, fish)	^•^ P14: be satisfied (cat)

P8 and P14 are not mandatory due to the lower frequency of less than 80% mentions in the normalization sample. The other propositions are counted as obligatory propositions (*N* = 12).

Examples of “peripheral propositons”: *The dog, no I mean the cat, steals the fish.* (self-correction) *Once I had a cat as a pet, too.* (meta-comment) *The woman eats the fish.* (thematically false utterance)

#### Subtest inferences

What has happened in the meantime?

Instruction: *Below you will read short texts about different events. “Please complete the missing line. What might have happened in between? Write or say a sentence that explains the outcome of the story. Please write complete sentences, not just keywords!”*

Example:

Family Meier is on the way to the summer vacation.

On the highway, traffic is getting heavier and heavier. _________________________________________________________ (missing line).

**Table:** They arrive at their vacation destination very late. Example of answers (translated from german)

3 points: bridging inference
*There was a traffic jam and they were stuck on the highway for a long time.*
2 points: bridging inference with linguistic-formal errors *stock in traffic on highway.*
1 point: thematically related key terms or ellipses (1 point): *Traffic jam*
0 points:
*Everyone likes to have a vacation.* (utterance does not fill the causal gap).
*They arrive late.* (repetition of a given sentence from the cloze).

## References

[B1] AbdulSabur N. Y. Xu Y. Liu S. Chow H. M. Baxter M. Carson J. (2014). Neural correlates and network connectivity underlying narrative production and comprehension: A combined fMRI and PET study. *Cortex* 57 107–127. 10.1016/j.cortex.2014.01.017 24845161

[B2] Alexander M. P. (2002). “Disorders of language after frontal lobe injury: Evidence for the neural mechanisms of disorders of language,” in *Principles of frontal lobe function*, eds Stuss D. T. Knight R. T. (Oxford: Oxford University Press), 159–167. 10.1093/acprof:oso/9780195134971.003.0010

[B3] Allain P. Togher L. Azouvi P. (2019). Social cognition and traumatic brain injury: Current knowledge. *Brain Inj.* 33 1–3. 10.1080/02699052.2018.1533143 30325238

[B4] Ardila A. (2012). “The executive functions in language and communication,” in *Cognition and acquired language disorders: An information processing approach*, eds Peach R. K. Shapiro L. P. (Amsterdam: Elsevier), 147–166. 10.1016/B978-0-323-07201-4.00016-7

[B5] Ardila A. Akhutina T. V. Mikadze Y. V. (2020). A.R. Luria’s contribution to studies of the brain organization of language. *RJTAO* 12 4–12. 10.14412/2074-2711-2020-1-4-12

[B6] Aschenbrenner S. Tucha O. Lange K. W. (2001). *Regensburger Wortflüssigkeitstest (RWT).* Göttingen: Hogrefe.

[B7] Bambini V. Bara B. G. (2012). “Neuropragmatics,” in *Handbook of pragmatics*, eds Östman J.-O. Verschueren J. (Amsterdam: John Benjamins Publishing Company), 1–21. 10.1075/hop.16.neu2

[B8] Bosco F. M. Gabbatore I. Angeleri R. Zettin M. Parola A. (2018). Do executive function and theory of mind predict pragmatic abilities following traumatic brain injury? An analysis of sincere, deceitful and ironic communicative acts. *J. Commun. Disord.* 75 102–117. 10.1016/j.jcomdis.2018.05.002 29887277

[B9] Braun C. M. Baribeau J. C. (1987). “Subclinical aphasia following closed head injury: A response to Sarno, Buonaguro, and Levita,” in *Proceedings of the clinical aphasiology conference*, Minneapolis, MN, 326–333.

[B10] Büttner J. (2014). *Sprache und Kognition: Diskurspragmatik und Textverarbeitung bei Exekutivstörungen.* Tübingen: Stauffenburg.

[B11] Büttner J. (2016). “Chapter 3. Neurolinguistic view into narrative processing,” in *Perspectives on narrativity and narrative perspectivization*, eds Igl N. Zeman S. (Amsterdam: John Benjamins Publishing Company), 63–88. 10.1075/lal.21.04but

[B12] Büttner J. (2017). *Neuropragmatik. Taxonomie von Kommunikationsstörungen und Diagnostik bei SHT. Aphasie und Verwandte Gebiete (Aphasie et Domaines Associés)* 1, 31–45.

[B13] Büttner J. (2018). *MAKRO: Screening zur Verarbeitung der Makrostruktur von Texten bei neurologischen Patienten.* Hofheim: NAT-Verlag.

[B14] Büttner J. Glindemann R. (2019). *Kognitive Kommunikationsstörungen. 1. Auflage.* Göttingen: Hogrefe. 10.1026/02818-000

[B15] Büttner-Kunert J. Anzenberger M. Müller V. P. Douglas J. (2021a). Bewertung des Gesprächsverhaltens nach Schädel-Hirn-Trauma mit dem La Trobe Communication questionnaire (LCQ): Erste Ergebnisse der deutschen Replikationsstudie an neurologisch gesunden Kontrollprobanden. *Sprache Stimme Gehör* 2 e7–e15. 10.1055/a-1158-3151

[B16] Büttner-Kunert J. Falkowska Z. Blöchinger S. (2021b). Is there an impact of theory of mind on narrative discourse production and social-communicative participation in people with traumatic brain injury and healthy individuals? *Neurol. Rehabil.* S1, page 11.

[B17] Büttner-Kunert J. Falkowska Z. Klonowski M. (2022). The MAKRO-screening – An assessment tool for discourse deficits in adults with dysexecutive symptoms following TBI. *Brain Inj.* 36 514–527. 10.1080/02699052.2022.2034957 35125058

[B18] Cannizzaro M. Coelho C. A. (2012). “Communication following executive dysfunction,” in *Cognition and acquired language disorders: An information processing approach*, eds Peach R. K. Shapiro L. P. (Amsterdam: Elsevier), 227–240. 10.1016/B978-0-323-07201-4.00020-9

[B19] Channon S. Watts M. (2003). Pragmatic language interpretation after closed head injury: Relationship to executive functioning. *Cogn. Neuropsychiatry* 8 243–260. 10.1080/135468000344000002 16571564

[B20] Christman Buckingham S. S. Sneed K. E. (2020). “Cognitive-communication disorder,” in *Encyclopedia of clinical neuropsychology*, eds Kreutzer J. S. DeLuca J. Caplan B. (Cham: Springer International Publishing), 1–8.

[B21] Coelho C. A. (2002). Story narratives of adults with closed head injury and non-brain-injured adults. *J. Speech Lang. Hear. Res.* 45 1232–1248. 10.1044/1092-4388(2002/099) 12546490

[B22] Coelho C. A. Lê K. Mozeiko J. Hamilton M. Tyler E. Krueger F. (2013). Characterizing discourse deficits following penetrating head injury: A preliminary model. *Am. J. Speech Lang. Pathol.* 22 S438–S448. 10.1044/1058-0360(2013/12-0076) 23695915 PMC7684633

[B23] Cohen J. (1988). *Statistical power analysis for the behavioral sciences*, 2nd Edn. Hillsdale, NJ: Lawrence Erlbaum Associates.

[B24] Cummings L. (ed.) (2017). *Research in clinical pragmatics.* Cham: Springer International Publishing. 10.1007/978-3-319-47489-2

[B25] Demir S. O. Altinok N. Aydin G. Köseoǧlu F. (2006). Functional and cognitive progress in aphasic patients with traumatic brain injury during post-acute phase. *Brain Inj.* 20 1383–1390. 10.1080/02699050601081844 17378230

[B26] DIMDI (ed.) (2012). *ICF – Internationale Klassifikation der Funktionsfähigkeit, Behinderung und Gesundheit. Unveränd. Nachdr.* Köln: DIMDI.

[B27] Douglas J. M. (2010a). Relation of executive functioning to pragmatic outcome following severe traumatic brain injury. *J. Speech Lang. Hear. Res.* 53 365–382. 10.1044/1092-4388(2009/08-0205) 20360462

[B28] Douglas J. M. (2010b). Using the La Trobe communication questionnaire to measure perceived social communication ability in adolescents with traumatic brain injury. *Brain Impair.* 11 171–182. 10.1375/brim.11.2.171

[B29] Douglas J. M. Bracy C. A. Snow P. C. (2007a). Exploring the factor structure of the La Trobe communication questionnaire: Insights into the nature of communication deficits following traumatic brain injury. *Aphasiology* 21 1181–1194. 10.1080/02687030600980950

[B30] Douglas J. M. Bracy C. A. Snow P. C. (2007b). Measuring perceived communicative ability after traumatic brain injury: Reliability and validity of the La Trobe communication questionnaire. *J. Head Trauma Rehabil.* 22 31–38. 10.1097/00001199-200701000-00004 17235229

[B31] Douglas J. M. Knox L. de Maio C. Bridge H. (2015). Improving communication-specific coping after traumatic brain injury: Evaluation of a new treatment using single-case experimental design. *Brain Impair.* 15, 190–201. 10.1017/BrImp.2014.25

[B32] Douglas J. M. Knox L. de Maio C. Bridge H. Drummond M. Whiteoak J. (2019). Effectiveness of communication-specific coping intervention for adults with traumatic brain injury: Preliminary results. *Neuropsychol. Rehabil.* 29 73–91. 10.1080/09602011.2016.1259114 27911168

[B33] Douglas J. M. O’flaherty C. A. Snow P. C. (2000). Measuring perception of communicative ability: The development and evaluation of the La Trobe communication questionnaire. *Aphasiology* 14 251–268. 10.1080/026870300401469

[B34] Drew L. B. Drew W. E. (2004). The contrecoup–coup phenomenon: A new understanding of the mechanism of closed head injury. *NCC* 1 385–390. 10.1385/NCC:1:3:385 16174940

[B35] Eichstaedt K. E. Kovatch K. Maroof D. A. (2013). A less conservative method to adjust for familywise error rate in neuropsychological research: The Holm’s sequential Bonferroni procedure. *Neurorehabilitation* 32 693–696. 10.3233/NRE-130893 23648625

[B36] Ellis C. Peach R. K. (2009). Sentence planning following traumatic brain injury. *Neurorehabilitation* 24 255–266. 10.3233/NRE-2009-0476 19458433

[B37] Falkowska Z. Büttner-Kunert J. (2020). Diskursstörungen und pragmatisches Profil nach Schädel-Hirn-Trauma (SHT). Multiperspektivische Einzelfallstudie mit Bezug auf Selbst- und Fremdeinschätzung. *Z. Neuropsychol*. 31, 169–170.

[B38] Falkowska Z. Heider N. Resch K. Royko J. Kunert B. (2021). Die Erhebung von kommunikativ-pragmatischen Fähigkeiten und Lebensqualität nach Schädel-Hirn-Trauma: Scoping-review. *Z. Neuropsychol*. 32, 181–193. 10.1024/1016-264X/a000336

[B39] Ferstl E. C. Guthke T. von Cramon D. Y. (2002). Text comprehension after brain injury: Left prefrontal lesions affect inference processes. *Neuropsychology* 16 292–308. 10.1037//0894-4105.16.3.292 12146677

[B40] Ferstl E. C. Neumann J. Bogler C. von Cramon D. Y. (2008). The extended language network: A meta-analysis of neuroimaging studies on text comprehension. *Hum. Brain Mapp.* 29 581–593. 10.1002/hbm.20422 17557297 PMC2878642

[B41] Ferstl E. C. Walther K. Guthke T. von Cramon D. Y. (2005). Assessment of story comprehension deficits after brain damage. *J. Clin. Exp. Neuropsychol.* 27 367–384. 10.1080/13803390490515784 15969358

[B42] Frith M. Togher L. Ferguson A. Levick W. Docking K. (2014). Assessment practices of speech-language pathologists for cognitive communication disorders following traumatic brain injury in adults: An international survey. *Brain Inj.* 28 1657–1666. 10.3109/02699052.2014.947619 25158134

[B43] Funder D. C. Ozer D. J. (2019). Evaluating effect size in psychological research: Sense and nonsense. *Adv. Methods Pract. Psychol. Sci.* 2 156–168. 10.1177/2515245919847202

[B44] Gabbatore I. Sacco K. Angeleri R. Zettin M. Bara B. G. Bosco F. M. (2015). Cognitive pragmatic treatment: A rehabilitative program for traumatic brain injury individuals. *J. Head Trauma Rehabil.* 30 E14–E28. 10.1097/HTR.0000000000000087 25310292

[B45] Gernsbacher M. A. (1990). *Language comprehension as structure building.* Hillsdale, NJ: Lawrence Erlbaum Associates. 10.21236/ADA221854

[B46] Grochmal-Bach B. Pachalska M. Markiewicz K. Tomaszewski W. Olszewski H. Pufal A. (2009). Rehabilitation of a patient with aphasia due to severe traumatic brain injury. *Med. Sci. Monit.* 15 CS67–CS76. 19333207

[B47] Hart T. Novack T. A. Temkin N. Barber J. Dikmen S. S. Diaz-Arrastia R. (2016). Duration of posttraumatic amnesia predicts neuropsychological and global outcome in complicated mild traumatic brain injury. *J. Head Trauma Rehabil.* 31 E1–E9. 10.1097/HTR.0000000000000210 26828710 PMC4738168

[B48] Hawley L. A. Newman J. K. (2010). Group interactive structured treatment (GIST): A social competence intervention for individuals with brain injury. *Brain Inj.* 24 1292–1297. 10.3109/02699052.2010.506866 20735320

[B49] Heilman K. M. Safran A. Geschwind N. (1971). Closed head trauma and aphasia. *J. Neurol. Neurosurg. Psychiatry* 34 265–269. 10.1136/jnnp.34.3.265 5571313 PMC1083462

[B50] Henry J. D. Crawford J. R. (2004). A meta-analytic review of verbal fluency performance in patients with traumatic brain injury. *Neuropsychology* 18 621–628. 10.1037/0894-4105.18.4.621 15506829

[B51] Hertrich I. Dietrich S. Ackermann H. (2020). The margins of the language network in the brain. *Front. Commun.* 5:519955. 10.3389/fcomm.2020.519955

[B52] Holland A. L. (1982). “When is aphasia aphasia? The problem with closed head injury,” in *Proceedings of the Clinical Aphasiology*, Minneapolis, MN, 345–349.

[B53] Huber W. Poeck K. Weniger D. Willmes K. (1983). *Aachener aphasie test.* Göttingen: Hogrefe.

[B54] Kerr C. (1995). Dysnomia following traumatic brain injury: An information-processing approach to assessment. *Brain Inj.* 9 777–796. 10.3109/02699059509008234 8605511

[B55] Kim J. Whyte J. Hart T. Vaccaro M. Polansky M. Coslett H. (2005). Executive function as a predictor of inattentive behavior after traumatic brain injury. *J. Int. Neuropsychol. Soc.* 11 434–445. 10.1017/S1355617705050563 16209424

[B56] Kintsch W. (2005). An overview of top-down and bottom-up effects in comprehension: The CI perspective. *Discourse Process.* 39 125–128. 10.1080/0163853X.2005.9651676

[B57] Krueger F. Grafman J. (2008). “The human prefrontal cortex stores structured event complexes,” in *Understanding events: How humans see, represent, and act on events*, eds Shipley T. F. Zacks J. M. (Cary, NC: Oxford University Press), 617–638. 10.1093/acprof:oso/9780195188370.003.0025

[B58] Laxe S. Zasler N. Selb M. Tate R. Tormos J. M. Bernabeu M. (2013). Development of the international classification of functioning, disability and Health core sets for traumatic brain injury: An international consensus process. *Brain Inj.* 27 379–387. 10.3109/02699052.2012.750757 23472615

[B59] Lê K. Coelho C. Mozeiko J. Krueger F. Grafman J. (2011). Measuring goodness of story narratives: Implications for traumatic brain injury. *Aphasiology* 25 748–760. 10.1080/02687038.2010.539696

[B60] Lê K. Coelho C. Mozeiko J. Krueger F. Grafman J. (2012). Predicting story goodness performance from cognitive measures following traumatic brain injury. *Am. J. Speech Lang. Pathol.* 21 115–125. 10.1044/1058-0360(2012/11-0114) 22294408

[B61] Lipton M. L. Gulko E. Zimmerman M. E. Friedman B. W. Kim M. Gellella E. (2009). Diffusion-tensor imaging implicates prefrontal axonal injury in executive function impairment following very mild traumatic brain injury. *Radiology* 252 816–824. 10.1148/radiol.2523081584 19567646

[B62] Luria A. R. (1970). *Traumatic aphasia: Its syndromes, psychology and treatment.* The Hague: Mouton. 10.1515/9783110816297

[B63] MacDonald S. (2015). *Cognitive-communication checklist for acquired brain injury (CCCABI)*. Guelph, ON: CCD Publishing. 10.1044/2021_AJSLP-20-00155 33871283

[B64] MacDonald S. (2017). Introducing the model of cognitive-communication competence: A model to guide evidence-based communication interventions after brain injury. *Brain Inj.* 31 1760–1780. 10.1080/02699052.2017.1379613 29064304

[B65] Maegele M. Lefering R. Sakowitz O. Kopp M. A. Schwab J. M. Steudel W.-I. (2019). The Incidence and management of moderate to severe head injury. *Deutsches Arzteblatt Int.* 116 167–173. 10.3238/arztebl.2019.0167 30995953 PMC6482369

[B66] Mar R. A. (2004). The neuropsychology of narrative: Story comprehension, story production and their interrelation. *Neuropsychologia* 42 1414–1434. 10.1016/j.neuropsychologia.2003.12.016 15193948

[B67] Marini A. Zettin M. Galetto V. (2014). Cognitive correlates of narrative impairment in moderate traumatic brain injury. *Neuropsychologia* 64 282–288. 10.1016/j.neuropsychologia.2014.09.042 25281884

[B68] Marini A. Zettin M. Bencich E. Bosco F. M. Galetto V. (2017). Severity effects on discourse production after TBI. *J. Neurolinguist.* 44 91–106. 10.1016/j.jneuroling.2017.03.005

[B69] Mason R. A. Just M. A. (2006). “Neuroimaging contributions to the understanding of discourse processes,” in *Handbook of psycholinguistics*, eds Traxler M. Gernsbacher M. A. (Amsterdam: Elsevier), 765–799. 10.1016/B978-012369374-7/50020-1

[B70] Mason R. A. Just M. A. (2011). Differentiable cortical networks for inferences concerning people’s intentions versus physical causality. *Hum. Brain Mapp.* 32 313–329. 10.1002/hbm.21021 21229617 PMC3049154

[B71] Mcdonald S. Honan C. Kelly M. Byom L. Rushby J. (2014). “Disorders of social cognition and social behaviour following sever TBI,” in *Social and communication disorders following traumatic brain injury*, 2nd Edn, eds McDonald S. Togher L. Code C. (London: Psychology Press), 119–159.

[B72] McDonald S. Togher L. Code C. (2014). “Traumatic brain injury: Basic features,” in *Social and communication disorders following traumatic brain injury*, 2nd Edn, eds McDonald S. Togher L. Code C. (London: Psychology Press).

[B73] Milders M. (2019). Relationship between social cognition and social behaviour following traumatic brain injury. *Brain Inj.* 33 62–68. 10.1080/02699052.2018.1531301 30325217

[B74] Mishra K. Purohit D. Sharma S. (2020). Montreal cognitive assessment score: A screening tool for cognitive function in traumatic brain injury (TBI) population. *J. Neurol. Neuromed.* 5 35–39. 10.29245/2572.942X/2020/3.1238

[B75] Mozeiko J. Le K. Coelho C. Krueger F. Grafman J. (2011). The relationship of story grammar and executive function following TBI. *Aphasiology* 25 826–835. 10.1080/02687038.2010.543983

[B76] Nasreddine Z. S. Phillips N. A. Bédirian V. Charbonneau S. Whitehead V. Collin I. (2005). The montreal cognitive assessment, MoCA: A brief screening tool for mild cognitive impairment. *J. Am. Geriatr. Soc.* 53 695–699. 10.1111/j.1532-5415.2005.53221.x 15817019

[B77] Neumann S. Quinting J. Rosenkranz A. De Beer C. Jonas K. Stenneken P. (2019). Quality of life in adults with neurogenic speech-language-communication difficulties: A systematic review of existing measures. *J. Communi. Disord.* 79 24–45. 10.1016/j.jcomdis.2019.01.003 30851625

[B78] Olver J. Ponsford J. Curran C. (1996). Outcome following traumatic brain injury: A comparison between 2 and 5 years after injury. *Brain Inj.* 10 841–848. 10.1080/026990596123945 8905161

[B79] Peach R. K. (2013). The cognitive basis for sentence planning difficulties in discourse after traumatic brain injury. *Am. J. Speech Lang. Pathol.* 22 S285–S297. 10.1044/1058-0360(2013/12-0081) 23695905

[B80] Ponsford J. L. Downing M. G. Olver J. Ponsford M. Acher R. Carty M. (2014). Longitudinal follow-up of patients with traumatic brain injury: Outcome at two, five, and ten years post-injury. *J. Neurotrauma* 31 64–77. 10.1089/neu.2013.2997 23889321

[B81] Ponsford J. L. Olver J. H. Curran C. (1995). A profile of outcome: 2 years after traumatic brain injury. *Brain Inj.* 9 1–10. 10.3109/02699059509004565 7874089

[B82] Power E. Weir S. Richardson J. Fromm D. Forbes M. MacWhinney B. (2020). Patterns of narrative discourse in early recovery following severe traumatic brain injury. *Brain Inj.* 34 98–109. 10.1080/02699052.2019.1682192 31661629 PMC8903041

[B83] Raukola-Lindblom M. Ljungqvist L. Kurki T. Tenovuo O. Laasonen M. (2021). Cognitive-linguistic outcome in moderate to severe diffuse axonal injury and association with fatigue. *Brain Inj.* 35 1674–1681. 10.1080/02699052.2021.2012824 35015614

[B84] Rickheit G. Strohner H. (eds.) (1985). *Inferences in text processing*. Amsterdam: North-Holland.

[B85] Rosenfeld J. V. McFarlane A. C. Bragge P. Armonda R. A. Grimes J. B. Ling G. S. (2013). Blast-related traumatic brain injury. *Lancet Neurol.* 12 882–893. 10.1016/S1474-4422(13)70161-3 23884075

[B86] Rosenkranz A. Kircher T. Nagels A. (2019). “Neuropsychological correlates underlying verbal fluency deficits in schizophrenia: The role of attention and executive function,” in *Interdisciplinary linguistic and psychiatric research on language disorders*, eds Erdeljac V. Sekulić Sović M. (Zagreb: Filozofski fakultet Sveučilišta u Zagrebu), 45–54. 10.17234/9789531758314.04

[B87] Roth C. Hardin K. (2021). “Cognitive communication disorders of mild traumatic brain injury,” in *Cognitive communication disorders*, 3rd Edn, ed. Kimbarow M. L. (San Diego, CA: Plural), 273–341.

[B88] Royko J. Büttner-Kunert J. (2021). Die Untersuchung von pragmatischen Fähigkeiten und kognitiver Empathiefähigkeit bei Morbus parkinson: Eine explorative kohortenstudie. *Forschung Sprache* 1, 68–79.

[B89] Sarno M. T. (1980). The nature of verbal impairment after closed head injury. *J. Nerv. Ment. Dis.* 168 685–692. 10.1097/00005053-198011000-00008 7441232

[B90] Sarno M. T. Buonaguro A. Levita E. (1986). Characteristics of verbal impairment in closed head injured patients. *Arch. Phys. Med. Rehabil.* 67 400–405. 2424401

[B91] Schwenkreis P. (2018). Versorgung und Outcome von Patienten mit Schädel-Hirn-Trauma. *Trauma Berufskrankh* 20 58–63. 10.1007/s10039-017-0271-9

[B92] Shao Z. Janse E. Visser K. Meyer A. S. (2014). What do verbal fluency tasks measure? Predictors of verbal fluency performance in older adults. *Front. Psychol.* 5:772. 10.3389/fpsyg.2014.00772 25101034 PMC4106453

[B93] Singer M. Lea R. B. (2012). “Inference and reasoning in discourse comprehension,” in *Cognitive pragmatics*, ed. Schmid H.-J. (Berlin: De Gruyter Mouton), 85–122. 10.1515/9783110214215.85

[B94] Snow P. C. Douglas J. M. Ponsfordoe J. L. (1999). Narrative discourse following severe traumatic brain injury: A longitudinal follow-up. *Aphasiology* 13 529–551. 10.1080/026870399401993

[B95] Snow P. Douglas J. Ponsford J. (1997). Conversational assessment following traumatic brain injury: A comparison across two control groups. *Brain Inj.* 11 409–429. 10.1080/026990597123403 9171927

[B96] Sohlberg M. M. MacDonald S. Byom L. Iwashita H. Lemoncello R. Meulenbroek P. (2019). Social communication following traumatic brain injury part I. State-of-the-art review of assessment tools. *Int. J. Speech Lang. Pathol.* 21 115–127. 10.1080/17549507.2019.1583280 30957561

[B97] Spreen O. Benton A. L. (1969). *Neurosensory centre comprehensive examination for aphasia: Manual of directions.* Victoria, BC: University of Victoria, Neuropsychology Laboratory.

[B98] Steel J. Elbourn E. Togher L. (2021). Narrative discourse intervention after traumatic brain injury. *Top. Lang. Disord.* 41 47–72. 10.1097/TLD.0000000000000241

[B99] Stronach S. T. Turkstra L. S. (2008). Theory of mind and use of cognitive state terms by adolescents with traumatic brain injury. *Aphasiology* 22 1054–1070. 10.1080/02687030701632187

[B100] Struchen M. A. Pappadis M. R. Mazzei D. K. Clark A. N. Davis L. C. Sander A. M. (2008). Perceptions of communication abilities for persons with traumatic brain injury: Validity of the La Trobe communication questionnaire. *Brain Inj.* 22 940–951. 10.1080/02699050802425410 19005886

[B101] Tate R. L. Pfaff A. (2000). Problems and pitfalls in the assessment of posttraumatic amnesia. *Brain Impair.* 1 116–129. 10.1375/brim.1.2.116

[B102] Teasdale G. Jennett B. (1974). Assessment of coma and impaired consciousness. *Lancet* 304 81–84. 10.1016/S0140-6736(74)91639-0 4136544

[B103] Thomsen I. V. (1975). Evaluation and outcome of aphasia in patients with severe closed head trauma. *J. Neurol. Neurosurg. Psychiatry* 38 713–718. 10.1136/jnnp.38.7.713 1159445 PMC1083253

[B104] Togher L. McDonald S. Code C. (2014a). “Social and communication disorders following traumatic brain injury,” in *Social and communication disorders following traumatic brain injury*, 2nd Edn, eds McDonald S. Togher L. Code C. (London: Psychology Press), 1–25.

[B105] Togher L. McDonald S. Coelho C. A. Byom L. (2014b). “Cognitive communication disability following TBI,” in *Social and communication disorders following traumatic brain injury*, 2nd Edn, eds McDonald S. Togher L. Code C. (London: Psychology Press), 89–118.

[B106] Togher L. McDonald S. Tate R. Power E. Ylvisaker M. Rietdijk R. (2010). *TBI express: A social communication training manual for people with traumatic brain injury (TBI) and their communication partners.* Sydney, NSW: Australian Society for the Study of Brain Impairment.

[B107] Togher L. McDonald S. Tate R. Rietdijk R. Power E. (2016). The effectiveness of social communication partner training for adults with severe chronic TBI and their families using a measure of perceived communication ability. *Neurorehabilitation* 38 243–255. 10.3233/NRE-151316 27030901

[B108] Turkstra L. S. Coelho C. Ylvisaker M. (2005). The use of standardized tests for individuals with cognitive-communication disorders. *Semin. Speech Lang.* 26 215–222. 10.1055/s-2005-922101 16278794

[B109] van Dijk T. A. (1980). *Macrostructures: An interdisciplinary study of global structures in discourse, interaction, and cognition.* Hillsdale, NJ: Erlbaum Associates.

[B110] van Dijk T. A. Kintsch W. (1983). *Strategies of discourse comprehension*, 3rd Edn. New York, NY: Academic Press.

[B111] Vas A. K. Chapman S. B. Cook L. G. (2015). Language impairments in traumatic brain injury: A window into complex cognitive performance. *Handbook Clin. Neurol.* 128 497–510. 10.1016/B978-0-444-63521-1.00031-5 25701903

[B112] Whiteside D. M. Kealey T. Semla M. Luu H. Rice L. Basso M. R. (2016). Verbal fluency: Language or executive function measure? *Appl. Neuropsychol.* 23 29–34. 10.1080/23279095.2015.1004574 26111011

[B113] Ylvisaker M. (2008). *Language intervention strategies in aphasia and related neurogenic communication disorders*, 5th Edn. Philadelphia, PA: Lippincott Williams & Wilkins.

[B114] Ylvisaker M. DeBonis D. (2000). Executive function impairment in adolescence. *Top. Lang. Disord.* 20 29–57. 10.1097/00011363-200020020-00005

[B115] Zacks J. M. Ferstl E. C. (2016). “Discourse comprehension,” in *Neurobiology of language*, eds Hickok G. Small S. L. (Amsterdam: Elsevier), 661–673. 10.1016/B978-0-12-407794-2.00053-5

[B116] Zeman S. (2016). “Perspectivization as a link between narrative micro- and macro-structure,” in *Perspectives on narrativity and narrative perspectivization*, eds Igl N. Zeman S. (Amsterdam: John Benjamins Publishing Company), 17–42. 10.1075/lal.21.02zem

[B117] Zwaan R. A. (1999). “Five dimensions of narrative comprehension: The event-indexing model,” in *Narrative comprehension, causality, and coherence: Essays in honor of Tom Trabasso*, eds Goldman S. R. Graesser A. C. van den Broek C. (Hillsdale, NJ: Lawrence Erlbaum Associates Publishers), 93–110.

